# AGING-RELATED VEGF IMPAIRS MUSCLE REGENERATION

**DOI:** 10.1093/geroni/igac059.1608

**Published:** 2022-12-20

**Authors:** Yori Endo, Charles Hwang, Yuteng Zhang, Ronald Neppl, Shailesh Argawal, Indranil Sinah

**Affiliations:** Harvard Medical School, Boston, Massachusetts, United States; Brigham and Women's Hospital, Boston, Massachusetts, United States; Brigham and Women's Hospital, Boston, Massachusetts, United States; Brigham and Women's Hospital, Boston, Massachusetts, United States; Brigham and Women's Hospital, Boston, Massachusetts, United States; Brigham and Women's Hospital, Boston, Massachusetts, United States

## Abstract

**Purpose:**

Aging is associated with frailty, a parameter that correlates with mortality and loss of muscle mass. The molecular mechanisms behind aging-associated impairment of muscle regeneration remain incompletely understood. We hypothesized VEGF-A with known role in angiogenesis and muscle progenitor differentiation to regulate regeneration in aged skeletal muscle.

**Methods:**

Young C57BL/6 (10 weeks old) and old C57BL/6 mice (24 months old) were subjected to muscle cryoinjury to induce regeneration. Quantifications of cross-sectional area (CSA) of regenerating myofibers were performed. Tibialis anterior muscle lysates was used for quantifying VEGF-A. To evaluate the role of VEGF in muscle regeneration, a similar experiment was performed on VEGFlo mice with a 75% decrease in VEGF-A activity and littermate controls. ML228, a hypoxia signaling activator that increases VEGF-A levels, was injected into young and old mice as well as VEGFlo and littermate controls.

**Results:**

Old mice exhibited marked reduction in the VEGF-A protein levels and regenerating myofiber CSA on DPI 10 (1250 vs. 833μm2, p<.001). Similarly, VEGFlo mice exhibited significantly smaller regenerating fiber CSA as compared to littermate controls on DPI 10 (541 vs. 238μm2, p=.0011). Pharmacological augmentation of VEGFA using ML228 increased muscle VEGF levels by 2 folds and skeletal muscle regeneration in both old mice (25% increase in regenerating fiber CSA, p<.01) and VEGFlo (20% increase in regenerating fiber CSA, p<.01) mice, but not young or littermate controls.

**Conclusions:**

Muscle regeneration declines with aging in correlation with loss of VEGFA levels within skeletal muscle. Supplementation of VEGFA represents a therapeutic target for sarcopenia.

